# Myasthenia Gravis as an Immune-Mediated Side Effect of Checkpoint Inhibitors

**DOI:** 10.7759/cureus.16316

**Published:** 2021-07-11

**Authors:** Caio T Heleno, Ala Mustafa, Nico A Gotera, Amy Tesar

**Affiliations:** 1 Internal Medicine, MercyOne North Iowa Medical Center, Mason City, USA

**Keywords:** ocular-myasthenia-gravis, checkpoints inhibitors, immune mediated side effect, pembrolizumab, acetylcholine receptor antibody

## Abstract

This is a case report of new-onset myasthenia gravis (MG) as an immune-mediated adverse event (irAE) related to the use of pembrolizumab in a patient with undifferentiated adenocarcinoma of the pancreato-biliary tract. Up to this moment, only 52 cases of new-onset MG have been related to immune checkpoint inhibitors (ICIs). She was diagnosed with ocular MG nearly three months after starting the use of the anti-programmed death-ligand 1 (PD-1) inhibitor. The diagnosis was confirmed by the presence of serum antibodies against the acetylcholine receptor and the patient was started on pyridostigmine with subsequent clinical improvement. The use of pembrolizumab was discontinued due to concomitant progression of the subjacent malignant disease.

## Introduction

Pembrolizumab, an immune checkpoint inhibitor (ICI) that inhibits programmed death-ligand 1 (PD-1), is part of the cancer treatment of the most frequent tumors of the US population like lung and breast and have been used in an increasing number of clinical indications in multiple malignancies exhibiting prolonged overall survival and durable responses. Severe immune-mediated adverse events (irAEs) are relatively common, occurring in up to 55% of cases; however, neurologic irAEs are uncommon and usually unspecific. Here we report a case of new-onset myasthenia gravis (MG) in a young woman with undifferentiated pancreaticobiliary adenocarcinoma.

## Case presentation

**Figure 1:**
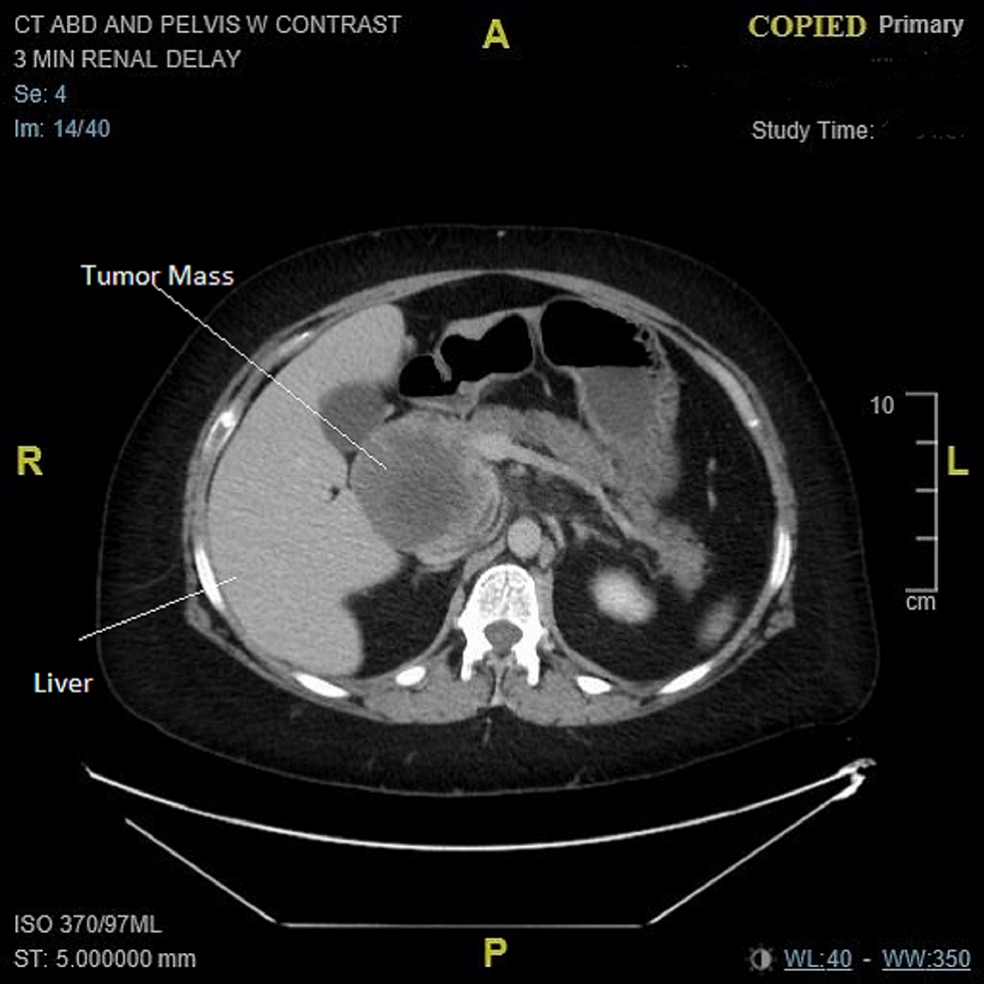
CT abdomen (axial plan): tumor mass in topography of head of the pancreas.

**Figure 2:**
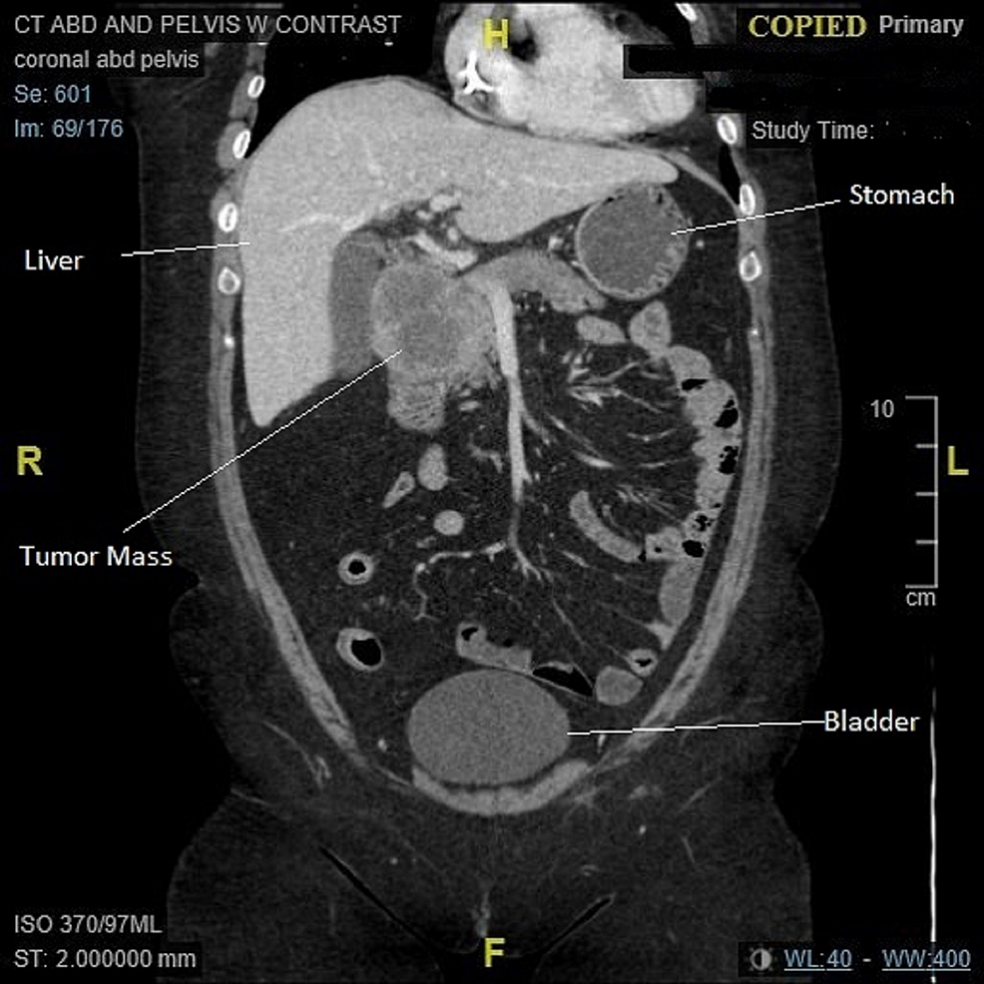
CT abdomen (coronal plan): tumor mass in topography of head of the pancreas.

A 43-year-old female with a past history of Crohn's disease status post colectomy 15 years ago and pulmonary sarcoidosis diagnosed at 35 years, presented to the emergency room on 07/2018 with complaints of severe abdominal pain and nausea. A computed tomography (CT) scan of the abdomen and pelvis showed a large mass adjacent to the head of the pancreas and the second portion of the duodenum (Figures 1, 2). Patient was admitted and blood work didn’t show any significant abnormality, with CEA (carcinoembryonic antigen) 0.7 ng/mL (NR: 0.0-5.4 ng/mL) and CA 19-9 (carbohydrate antigen 19-9) 13 U/mL (NR <= 34 U/mL). A positron emission tomography/magnetic resonance imaging (PET/MRI) showed a peri duodenal mass with a right-central hepatic lesion without distant metastasis. An endoscopic ultrasound pointed out a mass around the duodenum without any luminal lesion in the duodenum with biopsy positive for undifferentiated adenocarcinoma. Immunohistochemistry showed immunoreactive CK7 cells. CK20, CDX2 and P40 were negative suggesting origin in the gastrointestinal or pancreato-biliary tract. DNA mismatch repair test showed stable function within the tumor.

Patient was started on chemotherapy treatment with FOLFOX (5-fluorouracil/leucovorin and oxaliplatin), but after three cycles of treatment a PET/CT showed disease progression. The treatment was changed to gemcitabine and nab-paclitaxel that was used for five months without response, so she was started on concurrent chemoradiation with weekly gemcitabine. However, six months after finishing the chemoradiation treatment she was started once again on gemcitabine and Nab-paclitaxel due to the progression of disease. After two more months, PET/MRI showed progression of disease and she was started on FOLFIRI (5-fluorouracil/leucovorin and irinotecan). The regimen with FOLFIRI was used for 5 months and since there was disease progression, she was started on Pembrolizumab.

**Figure 3:**
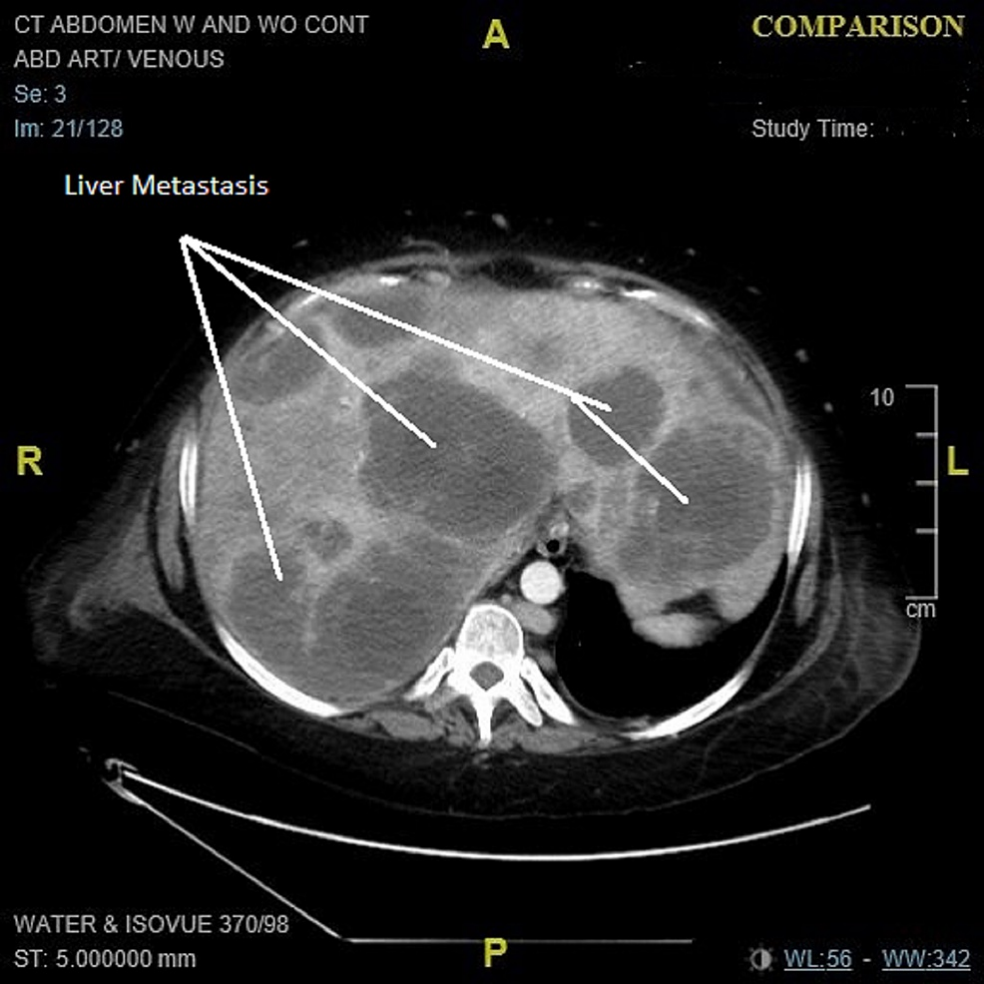
CT abdomen (axial plan): multiple liver metastasis.

**Figure 4:**
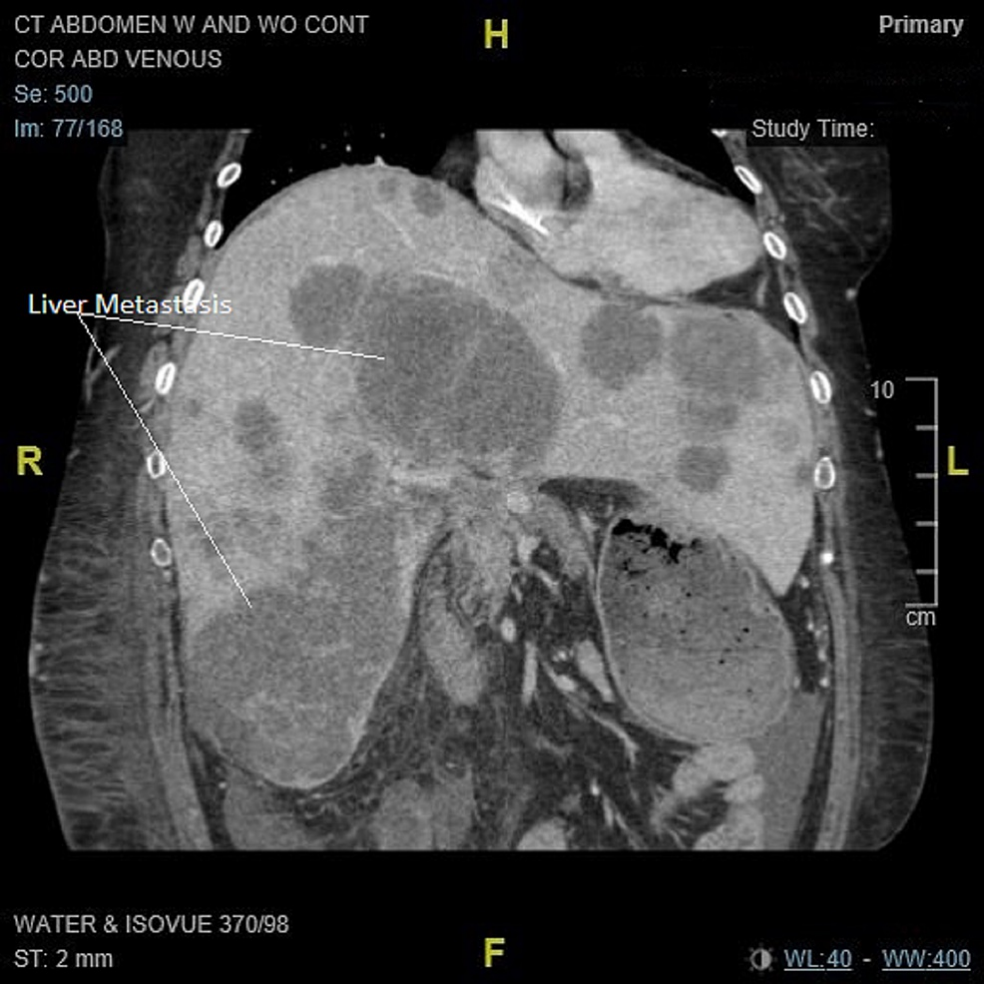
CT abdomen (coronal plan): multiple liver metastasis.

Patient was admitted to the emergency department three months after starting on pembrolizumab due to sudden onset of double vision and moderate headache. On physical examination, she was found to have diplopia with left hypertropia (vertical strabismus), Eastern Cooperative Oncology Group-performance status (ECOG-PS) of 2 without weakness, dyspnea or dysphagia. MRI of the brain didn't show any abnormality, but CT of abdomen and pelvis showed disease progression (Figures 3, 4). CA 19-9 of 158 U/mL (NR <=34 U/mL), with a normal CEA of 2.5 ng/ml, CPK was not checked. Due to neurologic symptoms and clinical suspicion of MG she was tested for anti-acetylcholine receptor (AChR) (Muscle) binding antibody, that showed an important elevation of 0.50 nmol/L (NR<=0.02 nmol/L). Patient was started on pyridostigmine 60mg TID, steroids were not used. Pembrolizumab was stopped due to disease progression. Patient had good control of the MG symptoms with use of acetylcholinesterase inhibitor. She died 12 months after the immunotherapy treatment was stopped.

## Discussion

Pembrolizumab has been associated with frequent and broad number of irAEs. Severe side effects are reported to happen in 10%-55% of cases according to the medication used, dose or combination with chemotherapy or second immunotherapy. However, neurologic irAEs are less frequent and usually mild and unspecific. Headache is responsible for 55% of the reported neurologic events [1-4]. The incidence of neurologic irAEs is 6.1% with the use of anti-PD-1 and 12% when there is a combination with anti-CTLA4 drugs. The overall incidence of neurologic symptoms on the use of anti-CTLA4 drugs is 3.8% [5]. MG have been reported in a very limited number of cases, a systematic review (SR) of the literature published by MD. Anderson Cancer Center in 11/2019 reported only 65 cases, 52 were newly developed onset MG and 11 were a flare of their preexisting MG [6].

The fluctuating weakness is the hallmark of myasthenia gravis, and repeated acts of a muscle group lead to its exhaustion. This gives rise to the classic presentation of muscle weakness, which worsens as the day progress or post-exercise. The muscle strength is restored with rest. Involvement of the ocular muscles is seen in more than 50% of affected individuals, with bulbar muscle involvement seen in about 15% and <5% involving the proximal limb muscles. The diagnostic approach of MG is focused on the clinical findings established by the history and typical examination signs, in addition to laboratory methods that aid in the confirmation diagnosis like electrophysiologic studies (repetitive nerve stimulation studies and single-fiber electromyography) and serologic tests for autoantibodies [7]. Serum antibodies against the acetylcholine receptor (AChR-Ab) are found in 93%, 88%, and 71% of individuals with moderate to severe generalized MG, mild generalized MG and ocular MG, respectively [1-4]. Besides that, they are highly specific for myasthenia gravis and there are virtually no false-positive results in healthy or disease-matched populations [8-10]. The safety data is still limited in patients with an underlying autoimmune disorder like our patient and is difficult to say that this could have acted as a predisposition factor.

According to SR published, 97% of the patients developed symptoms after a median of four weeks (from 1 to 16 weeks) of ICI initiation, 65% were males, 48% had metastatic melanoma with median age of 73 years [6,8]. Our patient started to have symptoms 16 weeks after starting the use of pembrolizumab and instead of the cases reported up to now, she was a young female with undifferentiated pancreaticobiliary adenocarcinoma.

## Conclusions

Pembrolizumab enhances the immune system, preventing the peripheral tolerance to cancer cells and downregulation of T-cell function. Besides irAEs are relatively common, neurologic adverse reactions are uncommon, especially specific diseases. Here we present an uncommon case of new-onset MG diagnosed 16 weeks after starting use of the medication. MG symptoms are unspecific, but diagnosis can be easily confirmed by serum antibodies against the acetylcholine receptor (AChR-Ab) that presents a high sensitivity and a negligible false positivity.
